# Field report: ambulance service in Ukraine during weaponized conflict

**DOI:** 10.1007/s11739-024-03728-y

**Published:** 2024-08-09

**Authors:** Beatrice Thielmann, Igor Zavgorodnii, Robin Schwarze, Victor Zabashta

**Affiliations:** 1 Institute of Occupational Medicine, Faculty of Medicine, Otto von Guericke University Magdeburg, Leipziger Str. 44, 39120 Magdeburg, Germany; 2 Department of Hygiene and Ecology No 2, Kharkiv National Medical University, Kharkiv, Ukraine; 3 Communal Non-Commercial Enterprise of the Kharkiv Regional Council “Center for Emergency Medical Care and Disaster Medicine in the Kharkiv Region”, Kharkiv, Ukraine

**Keywords:** Armed conflict, Ambulance service, Emergency medical service, Disaster medicine

## Abstract

Crises require changes to established structures, and this also applies to ambulance services. This case report addresses the Ukrainian ambulance service and the changes resulting from the armed conflict in Ukraine. The purpose of this article is to provide insight into the activities of the ambulance service of the Kharkiv region, the second-largest city in Ukraine. Kharkiv is still under heavy fire.

## Introduction

On February 24, 2022, Russian military units began to invade Ukraine. Currently, the conflict is continuing. The exact number of victims is unknown. Thirteen million Ukrainians have left their country since February 2022, and more than 6 million have returned [1–3]. Even in the early phase of the armed conflict, the infrastructure in eastern Ukraine (e.g., roads, buildings, and hospitals) was largely destroyed [4]. This was a potential hazard for ambulance service personnel because ambulances were sometimes unable to pass through the streets.

Numerous injured persons and challenges in the availability of medicines and medical equipment as well as potential gaps in staffing pose challenges to the healthcare system during times of crisis and [5], consequently, to  Ukraine's prehospital ambulance services.

This Point of View presents the prehospital rescue service of the city of Kharkiv. Kharkiv is located in northeastern Ukraine and is the second largest and most populous city after Kiev. About 1.5 million people live in this important scientific city of Ukraine [6]. It has significant cultural, industrial and scientific potential. Kharkiv is also the student and higher education center of Ukraine. The city is located near the border with the Russian Federation. Kharkiv is located 750 km from Moscow. The closest point to the border is the village of Goptovka, located 36 km from Kharkiv. From February to September 2022, Kharkiv was underground offensive, later heavy artillery fire for several months. About hundreds of civilians were killed and maimed [7]. It was not until the successful Ukrainian counteroffensive in September 2022 that the Kharkiv Oblast was largely liberated. In 2023, there were repeated missile and drone attacks. Kharkiv and the Kharkiv region are currently under Russian offensive again.

The aim of this case report is to describe the organizational structure of the Ukrainian ambulance service,  using the city of Kharkiv as an example, and its current situation during the armed conflict.

### Ambulance service of Ukraine and Kharkiv as example

In 2016, Ukraine’s emergency medical services system (EMS) was reformed nationwide and is controlled by the state through the Cabinet of Ministers of Ukraine. The Cabinet’s main tasks are to coordinate the Center for Emergency and Disaster Medicine, multidisciplinary hospitals, and EMS, and to set establish standards for the arrival of emergency medical teams to the scene.

The EMS is responsible for emergency care in a radius of approximately 8-18-10 km [4, 8]. Accessibility of 15 min to the patient within 15 min should be guaranteed. The number of ambulance teams is based on the population size, i.e., one team per 10,000  inhabitants in urban areas and 0.75 teams per 10,000  inhabitants in rural areas. The stations of the ambulance teams are classified into the following categories: category 3 includes: 2–5 ambulance vehicles, category 2 includes: 6–12 ambulance vehicles, and category 1 includes: 13 and or more ambulance vehicles. The team composition differs between medical and paramedic teams). The team leaders are personally responsible for the work on the team. The driver is not involved in medical treatment. After the reform in 2016, further qualification is possible. Field shearers can be trained as paramedics, and drivers can be trained as emergency medical technicians to assist  paramedics in emergency care. Paramedics have an educational level of at least a bachelor’s degree in the field of “health care” with a corresponding specialization. The duration of the program is three years for high school graduates or 4 years for secondary education. The qualification requirements for a paramedic are higher than for a field shearer because the training program is more focused on emergency care [4, 8]. Feldscherer is the highest non-academic training profession in the Ukrainian emergency ambulance service. The training lasts three years and is similar to that of a paramedic. Paramedics are trained for 3–4 years, depending on the basic school education (grades 9, 11) in the specialty of “general medicine”, qualification—paramedic. Unlike nurses, paramedics can work independently. On average, up to 60% of all emergency medical teams are composed of paramedics.

Control center dispatchers decide on the necessity need for an emergency response and alert the  emergency services teams [4, 8]. Typical emergency operations include, for example, syncope, convulsions, sudden shortness of breath, angina pectoris, hematemesis, acute/unclear abdomen, external bleeding/injury of various etiologies, heat stroke, hypothermia, asphyxia, acute infectious diseases, psychiatric disorders with danger to self and others, intoxication, animal bites, high-risk pregnancy, premature birth and gynecological bleeding [4, 8].

Although the population decreased (e.g., due to flight), the number of emergency operations remained unchanged. In Kharkiv and the Kharkiv region, 1000–1200 emergency missions are carried out every day. There are nine stations in the region with up to 10 teams each. In addition, there are a few private ambulance services, but they are much smaller. As expected, gunshot wounds top the list of emergency missions, followed by explosion injuries. The number of ambulance personnel and the number of teams have not changed. A shortage of professionals is not expected at this time, as the  profession is generally very well respected  by the population (even before the crises) and is very well paid. There are new recruitments when someone leaves the ambulance service. During the armed conflict since 2022, salaries have increased twice. In addition, all employees of the ambulance service receive cash bonuses and payment for missions to the front line. There is a psychological support service organized at the expense of voluntary associations.

In addition to the state-controlled ambulance service, there is also a private volunteer service. Since 2014, the aid organization Malteser Ukraine has been building a nationwide ambulance service with volunteer nonmedical rescue personnel. The volunteer ambulance service is still in the process of being established. With the beginning of the current armed conflict, it was established in twelve cities in Ukraine [9]. There is no government support. Thus, there is a lack of financial resources and a gap in materials such as medical aid supplies, surgical materials, dressing materials, infusions and resuscitation bags. Ambulance vehicles are needed, and there is a risk of a collapse of medical care during conflicts [9, 10].

### Challenges and changes to EMS since current weaponized conflict

**Fig. 1:**
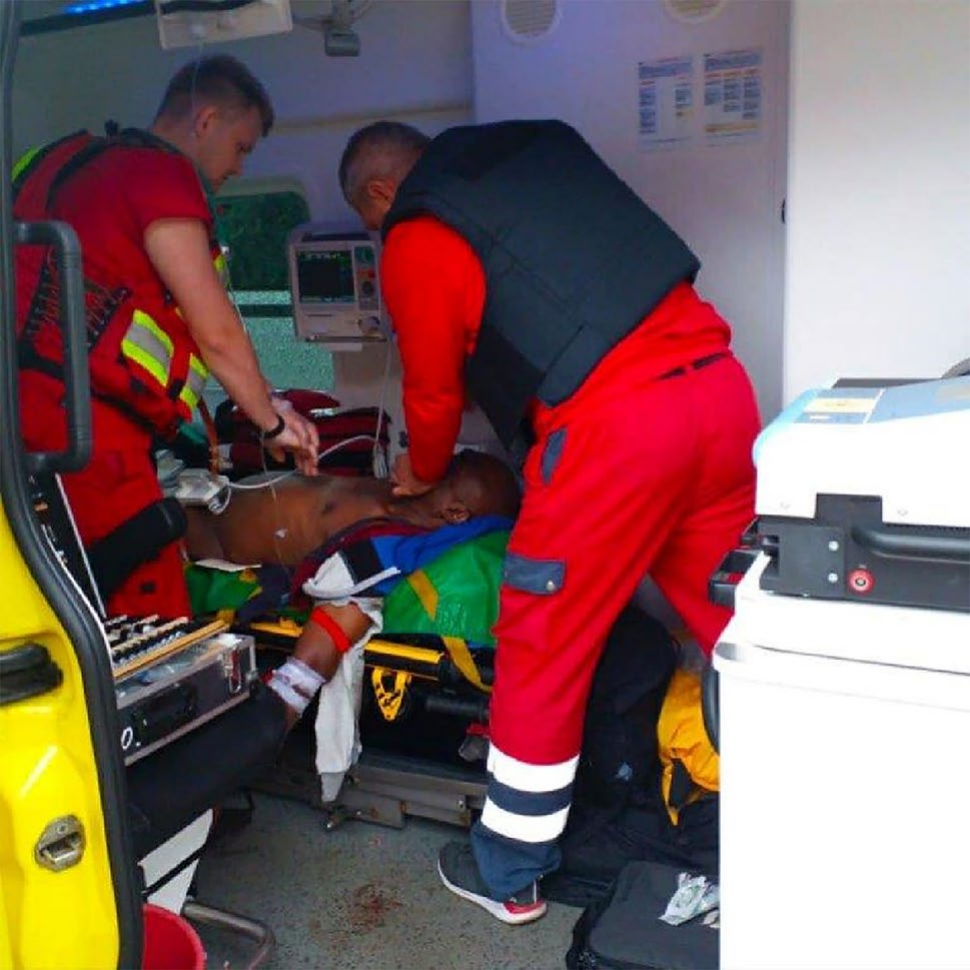
Wearing protective vests during operations; Photo/Photo rights: Victor Zabashta

**Fig. 2:**
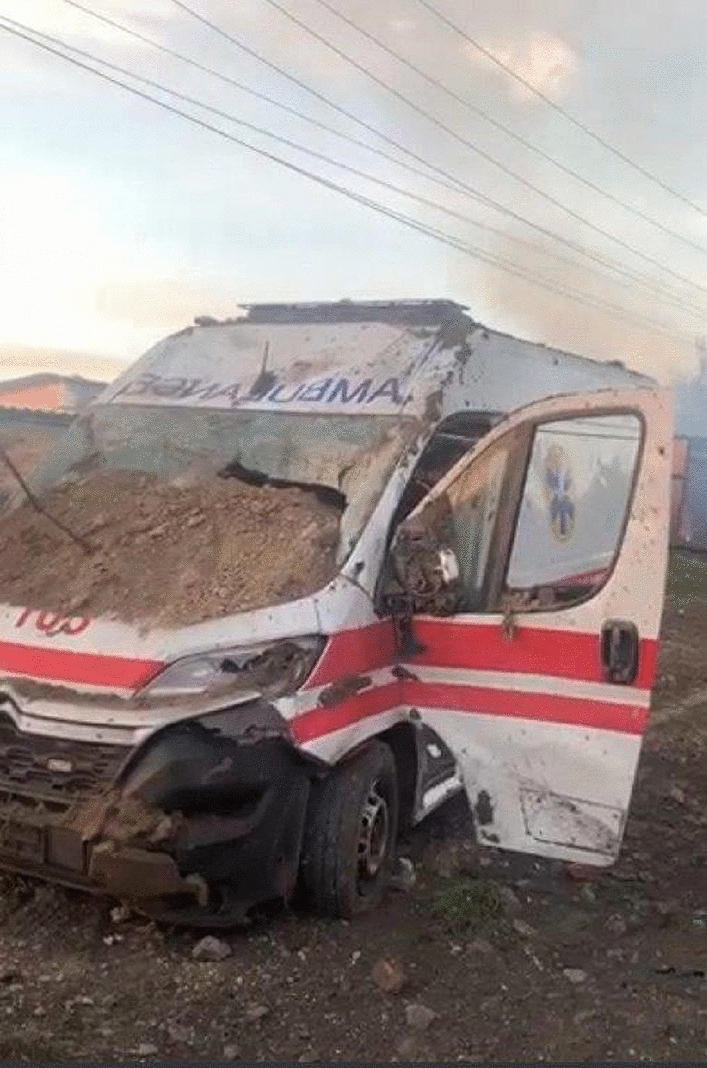
Ambulance vehicle destroyed during attack; Photo/Photo rights: Victor Zabashta

Since the beginning of the armed conflict the ambulance service has been operating continuously during the armed conflict. This is more or less feasible depending on the bombardment. During heavy shelling or airstrikes, for example, in the city of Kharkiv, the ambulance service teams disperse  throughout the city in order to reach the various sites of operation more quickly. These are usually located near metro stations or in cement-built stations. If the danger to life is particularly high, the ambulance service teams retreat to neighboring villages or towns. Ambulance services near the front lines are particularly risky. EMS personnel risk their lives every day to help others, whether civilians or soldiers. Emergency calls put them back in harm's way. This  is true in all regions under the Ukrainian flag.  At best, they wear protective vests because they are under fire. Since the armed conflict, there have been no changes in the organization and structure of emergency medical services. The changes have only affected the location, i.e. the location of the ambulance teams in the Kharkiv region. The changes occurred because of shelling, destruction and loss of material assets. Since the outbreak of the conflict, the protocols of emergency medical services have been updated, with special emphasis on tactical medicine (similar to the work of paramedics). What’s new is that the prehospital ambulance service also take care for wounded military personnel, e.g. by working together with the military’s medical services during transportation and evacuation. Ambulance crews have completed self-defense courses. There are no weapons for self-defense (Figs. 1, 2).

The ambulance service also organized the evacuation operations of patients. In March 2022, 22 ambulance service teams evacuated more than 100 people from Kharkiv’s Balakliia “(eastern Kharkiv Oblast). On April 22, 615 mentally disordered patients were evacuated from a hospital to Striletscha by emergency vehicles within 3 days. Emergency medical personnel are actively involved in evacuating the population. No further details can be provided at present.

## Conclusions

With remarkable engagement and at risk of their own lives, Ukrainian emergency medical services go about their daily work routines even in times of armed conflict. As expected, gunshot wounds lead to emergency operations.

Armed conflicts have disastrous consequences for the physical and mental health of all people involved (those directly involved and the civilian population) [11–14]. Armed conflicts have caused significant loss of life. Armed conflicts are the cause of high rates of disability and increase the prevalence of infectious diseases. Human rights violations  have also been observed [6]. Unfortunately, these challenges persist long after the armed conflict  has ended [15].

## Data Availability

Not applicable.
